# Big Data Analytics in Immunology: A Knowledge-Based Approach

**DOI:** 10.1155/2014/437987

**Published:** 2014-06-22

**Authors:** Guang Lan Zhang, Jing Sun, Lou Chitkushev, Vladimir Brusic

**Affiliations:** ^1^Department of Computer Science, Metropolitan College, Boston University, Boston, MA 02215, USA; ^2^Cancer Vaccine Center, Dana-Farber Cancer Institute, Harvard Medical School, Boston, MA 02115, USA

## Abstract

With the vast amount of immunological data available, immunology research is entering the big data era. These data vary in granularity, quality, and complexity and are stored in various formats, including publications, technical reports, and databases. The challenge is to make the transition from data to actionable knowledge and wisdom and bridge the knowledge gap and application gap. We report a knowledge-based approach based on a framework called KB-builder that facilitates data mining by enabling fast development and deployment of web-accessible immunological data knowledge warehouses. Immunological knowledge discovery relies heavily on both the availability of accurate, up-to-date, and well-organized data and the proper analytics tools. We propose the use of knowledge-based approaches by developing knowledgebases combining well-annotated data with specialized analytical tools and integrating them into analytical workflow. A set of well-defined workflow types with rich summarization and visualization capacity facilitates the transformation from data to critical information and knowledge. By using KB-builder, we enabled streamlining of normally time-consuming processes of database development. The knowledgebases built using KB-builder will speed up rational vaccine design by providing accurate and well-annotated data coupled with tailored computational analysis tools and workflow.

## 1. Introduction

**Figure 1:**
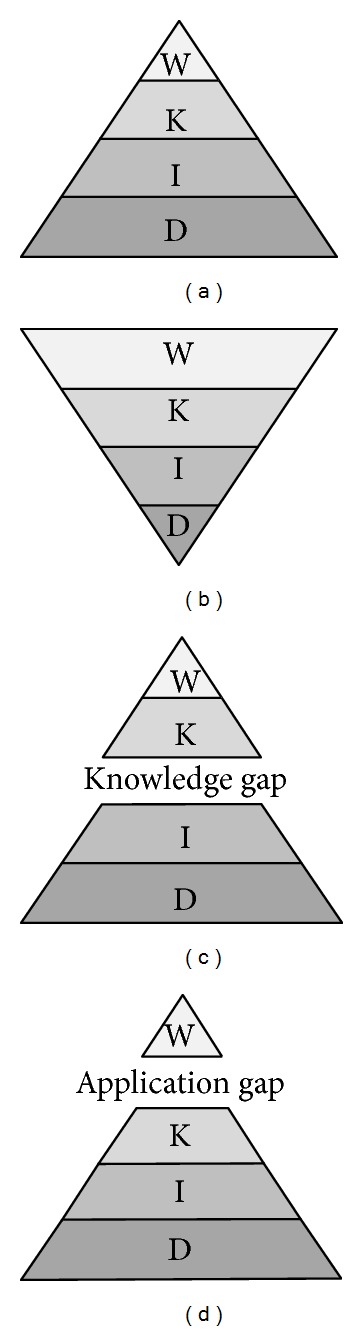
The DIKW hierarchy. (a) The relative quantities of data, information, knowledge, and wisdom. (b) The relative acquisition cost of the different layers. (c) The gap between data and knowledge and (d) the gap between knowledge and wisdom.

Data represent the lowest level of abstraction and do not have meaning by themselves. Information is data that has been processed so that it gives answers to simple questions, such as “what,” “where,” and “when.” Knowledge represents the application of data and information at a higher level of abstraction, a combination of rules, relationships, ideas, and experiences, and gives answers to “how” or “why” questions. Wisdom is achieved when the acquired knowledge is applied to offer solutions to practical problems. The data, information, knowledge, and wisdom (DIKW) hierarchy summarizes the relationships between these levels, with data at its base and wisdom at its apex and each level of the hierarchy being an essential precursor to the levels above (Figure 1(a)) [1, 2]. The acquisition cost is lowest for data acquisition and highest for knowledge and wisdom acquisition (Figure 1(b)).

In immunology, for example, a newly sequenced molecular sequence without functional annotation is a data point, information is gained by annotating the sequence to answer questions such as which viral strain it originates from, knowledge may be obtained by identifying immune epitopes in the viral sequence, and the design of a peptide-based vaccine using the epitopes represents the wisdom level. Overwhelmed by the vast amount of immunological data, to make the transition from data to actionable knowledge and wisdom and bridge the knowledge gap and application gap, we are confronted with several challenges. These include asking the “right questions,” handling unstructured data, data quality control (garbage in, garbage out), integrating data from various sources in various formats, and developing specialized analytics tools with the capacity to handle large volume of data.

The human immune system is a complex system comprising the innate immune system and the adaptive immune system. There are two branches of adaptive immunity, humoral immunity effected by the antibodies and cell-mediated immunity effected by the T cells of the immune system. In humoral immunity, B cells produce antibodies for neutralization of extracellular pathogens and their antigens that prevent the spread of infection. The activation of B cells and their differentiation into antibody-secreting plasma cells is triggered by antigens and usually requires helper T cells [3]. B cells identify antigens through B-cell receptors, which recognize discrete sites on the surface of target antigens called B-cell epitopes [4].

Cellular immunity involves the activation of phagocytes, antigen-specific cytotoxic T-lymphocytes (CTLs), and the release of various cytokines in response to pathogens and their antigens. T cells identify foreign antigens through their T-cell receptors (TCRs), which interact with a peptide antigen in complex with a major histocompatibility complex (MHC) molecule in conjunction with CD4 or CD8 coreceptors [5, 6]. Peptides that induce immune responses, when presented by MHC on the cell surface for recognition by T cells, are called T-cell epitopes. CD8+ T cells control infection through direct cytolysis of infected cells and through production of soluble antiviral mediators. This function is mediated by linear peptide epitopes presented by MHC class I molecules. CD4+ T cells recognize epitopes presented by MHC class II molecules on the surface of infected cells and secrete lymphokines that stimulate B cells and cytotoxic T cells. The Immune Epitope Database (IEDB) [7] hosts nearly 20,000 T-cell epitopes as of Feb. 2014.

The recognition of a given antigenic peptide by an individual immune system depends on the ability of this peptide to bind one or more of the host's human leukocyte antigens (HLA-human MHC). The binding of antigenic peptides to HLA molecules is the most selective step in identifying T-cell epitopes. There is a great diversity of HLA genes with more than 10,000 known variants characterized as of Feb. 2014 [8]. To manage this diversity, the classification of HLA into supertypes was proposed to describe those HLA variants that have small differences in their peptide-binding grooves and share similar peptide-binding specificities [9, 10]. Peptides that can bind multiple HLA variants are termed “promiscuous peptides.” They are suitable for the design of epitope-based vaccines because they can interact with multiple HLA within human populations.

**Figure 2:**
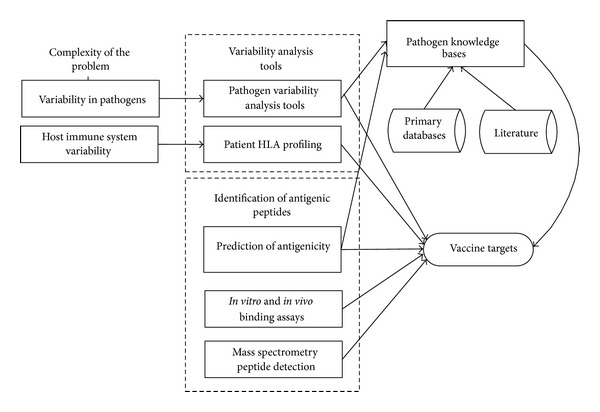
The process of rational vaccine discovery using knowledge-based systems. The design of broadly protective peptide-based vaccines involves identification and selection of vaccine targets composed of conserved T-cell and B-cell epitopes that are broadly cross-reactive to pathogen subtypes and protective of a large host population.

The concept of reverse vaccinology supports identification of vaccine targets by large-scale bioinformatics screening of entire pathogenic genomes followed by experimental validation [11]. Using bioinformatics analysis to select a small set of key wet-lab experiments for vaccine design is becoming a norm. The complexity of identification of broadly protective vaccine targets arises from two principal sources, the diversity of pathogens and the diversity of human immune system. The design of broadly protective peptide-based vaccines involves the identification and selection of vaccine targets composed of conserved T-cell and B-cell epitopes that are broadly cross-reactive to viral subtypes and protective of a large host population (Figure 2).

Fuelled by the breakthroughs in genomics and proteomics and advances in instrumentation, sample processing, and immunological assays, immunology research is entering the big data era. These data vary in granularity, quality, and complexity and are stored in various formats, including publications, technical reports, and databases. Next generation sequencing technologies are shifting the paradigm of genomics and allowing researchers to perform genome-wide studies [12]. It was estimated that the amount of publically available genomic data will grow from petabytes (10^15^) to exabytes (10^18^) [13]. Mass spectrometry (MS) is the method for detection and quantitation of proteins. The technical advancements in proteomics support exponential growth of the numbers of characterized protein sequences. It is estimated that more than 2 million protein variants make the posttranslated human proteome in any human individual [14]. Capitalizing on the recent advances in immune profiling methods, the Human Immunology Project Consortium (HIPC) is creating large data sets on human subjects undergoing influenza vaccination or who are infected with pathogens including influenza virus, West Nile virus, herpes zoster, pneumococcus, and the malaria parasite [15]. Systems biology aims to study the interactions between relevant molecular components and their changes over time and enable the development of predictive models. The advent of technological breakthroughs in the fields of genomics, proteomics, and other “omics” is catalyzing advances in systems immunology, a new field under the umbrella of system biology [16]. The synergy between systems immunology and vaccinology enables rational vaccine design [17].

Big data describes the environment where massive data sources combine both structured and unstructured data so that the analysis cannot be performed using traditional database and analytical methods. Increasingly, data sources from literature and online sources are combined with the traditional types of data [18] for summarization of complex information, extraction of knowledge, decision support, and predictive analytics. With the increase of the data sources, both the knowledge and application gaps (Figures 1(c) and 1(d)) keep widening and the corresponding volumes of data and information are rapidly increasing. We describe a knowledge-based approach that helps reduce the knowledge and application gaps for applications in immunology and vaccinology.

## 2. Materials and Methods

**Figure 3:**
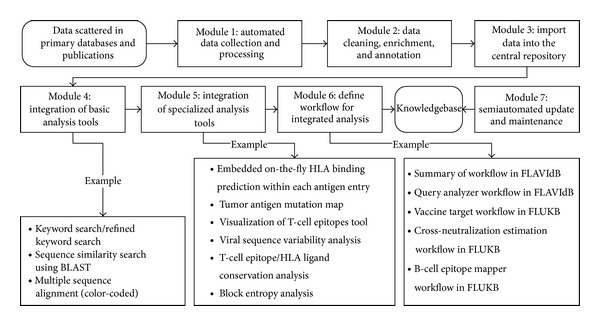
The structure of KB-builder.

In the big data era, knowledge-based systems (KBSs) are emerging as knowledge discovery platforms. A KBS is an intelligent system that employs a computationally tractable knowledgebase or repository in order to reason upon data in a targeted domain and reproduce expert performance relative to such reasoning operations [19]. The goal of a KBS is to increase the reproducibility, scalability, and accessibility of complex reasoning tasks [20]. Some of the web-accessible immunological databases, such as Cancer Immunity Peptide Database that hosts four static data tables containing four types of tumor antigens with defined T-cell epitopes, focus on cataloging the data and information and pay little attention to the integration of analysis tools [21, 22]. Most recent web-accessible immunological databases, such as Immune Epitope Database (IEDB) that catalogs experimentally characterized B-cell and T-cell epitopes and data on MHC binding and MHC ligand elution experiments, started to integrate some data analysis tools [7, 23]. To bridge the knowledge gap between immunological information and knowledge, we need KBSs that tightly integrate data with analysis tools to enable comprehensive screening of immune epitopes from a comprehensive landscape of a given disease (such as influenza, flaviviruses, or cancer), the analysis of crossreactivity and crossprotection following immunization or vaccination, and prediction of neutralizing immune responses. We developed a framework called KB-builder to facilitate data mining by enabling fast development and deployment of web-accessible immunological data knowledge warehouses. The framework consists of seven major functional modules (Figure 3), each facilitating a specific aspect of the knowledgebase construction process. The KB-builder framework is generic and can be applied to a variety of immunological sequence datasets. Its aim is to enable the development of a web-accessible knowledgebase and its corresponding analytics pipeline within a short period of time (typically within 1-2 weeks), given a set of annotated genetic or protein sequences.

The design of a broadly protective peptide-based vaccine against viral pathogens involves the identification and selection of vaccine targets composed of conserved T-cell and B-cell epitopes that are broadly cross-reactive to a wide range of viral subtypes and are protective in a large majority of host population (Figure 2). The KB-builder facilitates a systematic discovery of vaccine targets by enabling fast development of specialized bioinformatics KBS that tightly integrate the content (accurate, up-to-date, and well-organized antigen data) with tailored analysis tools.

**Figure 4:**
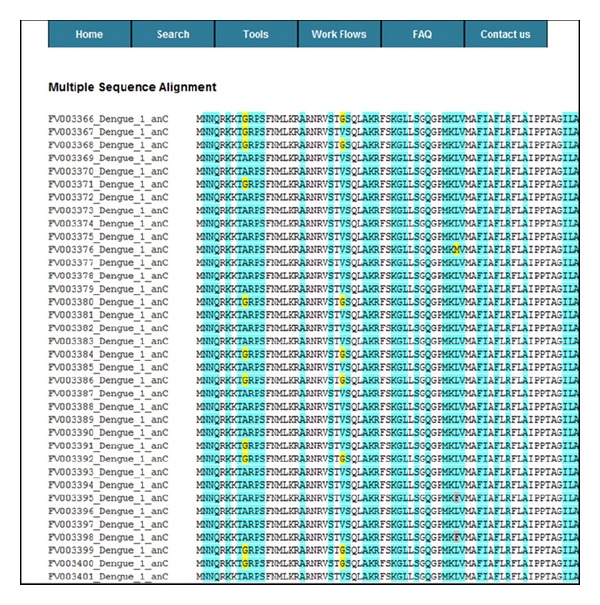
A screenshot of the result page generated by the color-coded MSA tool implemented in the FLAVIdB. The residues are color-coded by frequency: white (100%), cyan (second most frequent), yellow (third most frequent residues), gray (fourth most frequent residues), green (fifth most frequent residues), purple (sixth most frequent residues), and blue (everything less frequent than the sixth most frequent residues).

**Figure 5:**
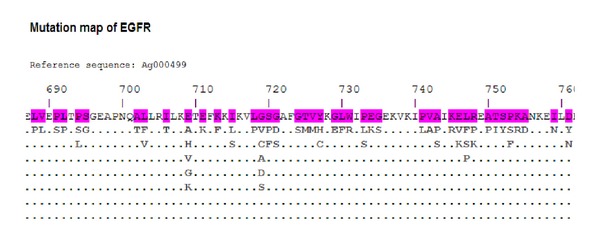
A screenshot of mutation map of tumor antigen epidermal growth factor receptor (EGFR) in TANTIGEN. The numbers are the amino acid positions in the antigen sequence and the top amino acid sequence is the reference sequence of EGFR. The highlighted amino acids in the reference sequences are positions where point mutations took place. Clicking on the amino acids below the point mutation positions links to the mutated sequence data table.

The input to KB-builder is data scattered across primary databases and scientific literature (Figure 3). Module 1 (data collection and processing module) performs automated data extraction and initial transformations. The raw antigen data (viral or tumor) consisting of protein or nucleotide sequences, or both, and their related information are collected from various sources. The collected data are then reformatted and organized into a unified XML format. Module 2 (data cleaning, enrichment, and annotation module) deals with data incompleteness, inconsistency, and ambiguities due to the lack of submission standards in the online primary databases. The semiautomated data cleaning is performed by domain experts to ensure data quality, completeness, and redundancy reduction. Semiautomated data enrichment and annotation are performed by the domain experts further enhancing data quality. The semiautomation involves automated comparison of new entries to the entries already processed within the KB and comparison of terms that are entered into locally implemented dictionaries. Terms that match the existing record annotations and dictionary terms are automatically processed. New terms and new annotations are inspected by a curator and if in error they are corrected, or if they represent novel annotations or terms they are added to the knowledgebase and to the local dictionaries. Module 3 (the import module) performs automatic import of the XML file into the central repository. Module 4 (the basic analysis toolset) facilitates fast integration of common analytical tools with the online antigen KB. All our knowledgebases have the basic keyword search tools for locating antigens and T-cell epitopes or HLA ligands. The advanced keyword search tool was included in FLAVIdB, FLUKB, and HPVdB, where users further restrict the search by selecting virus species, viral subtype, pathology, host organism, viral strain type, and several other filters. Other analytical tools include sequence similarity search enabled by basic local alignment search tool (BLAST) [24] and color-coded multiple sequence alignment (MSA) tool [25] on user-defined sequence sets as shown in Figure 4. Module 5 (the specialized analysis toolset) facilitates fast integration of specialized analysis tools designed according to the specific purpose of the knowledgebase and the structural and functional properties of the source of the sequences. To facilitate efficient antigenicity analysis, in every knowledgebase and within each antigen entry, we embedded a tool that performs on-the-fly binding prediction to 15 frequent HLA class I and class II alleles. In TANTIGEN, an interactive visualization tool, mutation map, has been implemented to provide a global view of all mutations reported in a tumor antigen.  Figure 5 shows a screenshot of mutation map of tumor antigen epidermal growth factor receptor (EGFR) in TANTIGEN. In TANTIGEN and HPVdB, a T-cell epitope visualization tool has been implemented to display epitopes in all isoforms of a tumor antigen or sequences of a HPV genotype. The B-cell visualization tool in FLAVIdB and FLUKB displays neutralizing B-cell epitope positions on viral protein three-dimensional (3D) structures [26, 27]. To analyze viral sequence variability, given a MSA of a set of sequences, a tool was developed to calculate Shannon entropy at each alignment position. To identify conserved T-cell epitopes that cover the majority of viral population, we developed and integrated block entropy analysis tool in FLAVIdB and FLUKB to analyze peptide conservation and variability. We developed a novel sequence logo tool, BlockLogo, optimized for visualization of continuous and discontinuous motifs, fragments [28, 29]. When paired with the HLA binding prediction tool, BlockLogo is a useful tool for rapid assessing of immunological potential of selected regions in a MSA, such as alignments of viral sequences or tumor antigens.

A workflow is an automated process that takes a request from the user, performs complex analysis by combining data and tools preselected for common questions, and produces a comprehensive report [30]. Module 6 (workflow for integrated analysis to answer meaningful questions) automates the consecutive execution of multiple analysis steps, which researchers usually would have to perform manually, to answer complex sequential questions. Two workflow types, the summary workflow and the query analyzer workflow, were implemented in FLAVIdB. Three workflow types, the vaccine target workflow, the crossneutralization estimation workflow, and B-cell epitope mapper workflow, were implemented in FLUKB. Module 7 (semiautomated update and maintenance of the databases) employs a semiautomated approach to maintain and update the databases.

## 3. Results and Discussion

Using the KB-builder, we built several immunovaccinology knowledgebases including TANTIGEN: Tumor T-cell Antigen Database (http://cvc.dfci.harvard.edu/tadb/), FLAVIdB: Flavivirus Antigen Database [31], HPVdB: Human Papillomavirus T-cell Antigen Database [32], FLUKB: Flu Virus Antigen Database (http://research4.dfci.harvard.edu/cvc/flukb/), Epstein-Barr Virus T-cell Antigen Database (http://research4.dfci.harvard.edu/cvc/ebv/), and Merkel Cell Polyomavirus Antigen Database (http://cvc.dfci.harvard.edu/mcv/). These knowledgebases combine virus and tumor antigenic data, specialized analysis tools, and workflow for automated complex analyses focusing on applications in immunology and vaccinology.

The Human Papillomavirus T-cell Antigen Database (HPVdB) contains 2781 curated antigen entries of antigenic proteins derived from 18 genotypes of high-risk HPV and 18 genotypes of low-risk HPV. It also catalogs 191 verified T-cell epitopes and 45 verified HLA ligands. The functions of the data mining tools integrated in HPVdB include antigen and epitope/ligand search, sequence comparison using BLAST search, multiple alignments of antigens, classification of HPV types based on cancer risk, T-cell epitope prediction, T-cell epitope/HLA ligand visualization, T-cell epitope/HLA ligand conservation analysis, and sequence variability analysis.

**Figure 6:**
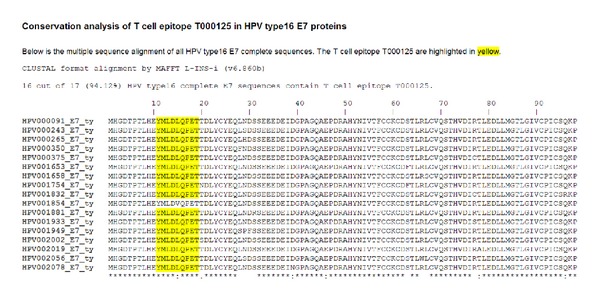
A screenshot of the conservation analysis result page of T-cell epitope E7_11–19_ in HPVdB.

HPV regulatory proteins E6 and E7 proteins are often studied for immune-based therapies as they are constitutively expressed in HPV-associated cancer cells. First, the prediction of A∗0201 binding peptides (both 9-mers and 10-mers) of HPV16 E6 and E7 proteins was performed computationally. Based on the prediction results, 21 peptides were synthesized and ten of them were identified as binders using an A∗0201 binding assay. The ten A∗0201-binding peptides were further tested for immune recognition in peripheral blood mononuclear cells isolated from six A∗0201-positive healthy donors using interferon *γ* (IFN *γ*) ELISpot assay. Two peptides, E7_11–19_ and E6_29–38_, elicited spot-forming-unit numbers 4-5-fold over background in one donor. Finally, mass spectrometry was used to validate that peptide E7_11–19_ is naturally presented on HPV16-transformed, A∗0201-positive cells. Using the peptide conservation analysis tool embedded in HPVdB, we answered the question how many HPV strains contain this epitope. The epitope E7_11–19_ is conserved in 16 of 17 (94.12% conserved) HPV16 E7 complete sequences (Figure 6). A single substitution mutation L15V in HPV001854 (UniProt ID: C0KXQ5) resulted in the immune escape. Among the 35 HPV16 cervical cancer samples we analyzed, only a single sample contained the HPV001854 sequence variant. The conserved HPV T-cell epitopes displayed by HPV transformed tumors such as E7_11–19_ may be the basis of a therapeutic T-cell based cancer vaccine. This example shows the combination of bioinformatics analysis and experimental validation leading to identification of suitable vaccine targets [33, 34].

Flaviviruses, such as dengue and West Nile viruses, are NIAID Category A and B Priority Pathogens. We developed FLAVIdB that contains 12,858 entries of flavivirus antigen sequences, 184 verified T-cell epitopes, 201 verified B-cell epitopes, and 4 representative molecular structures of the dengue virus envelope protein [31]. The data mining system integrated in FLAVIdB includes tools for antigen and epitope/ligand search, sequence comparison using BLAST search, multiple alignments of antigens, variability and conservation analysis, T-cell epitope prediction, and characterization of neutralizing components of B-cell epitopes. A workflow is an automated process that takes a request from the user, performs complex analysis by combining data and tools preselected for common questions, and produces a comprehensive report to answer a specific research question. Two predefined analysis workflow types, summary workflow and query analyzer workflow, were implemented in FLAVIdB [31].

**Figure 7:**
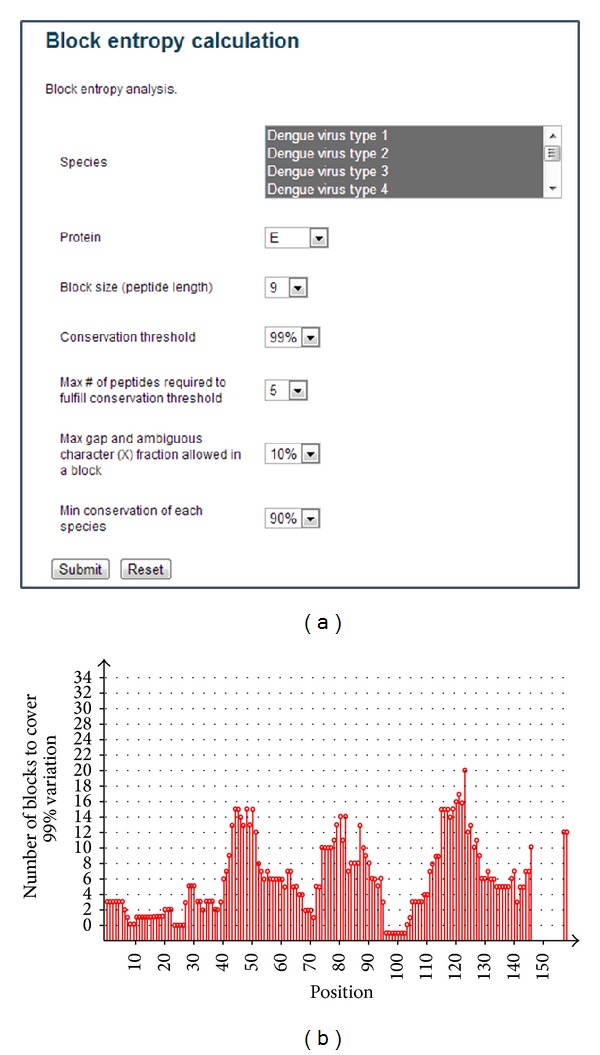
Block entropy analysis of envelope proteins of dengue subtypes 1–4 in the FLAVIdB. (a) A screenshot of the input page of block entropy analysis in the FLAVIdB. (b) The number of blocks needed to cover 99% of the sequences variation. *x*-axis is the starting positions of blocks and *y*-axis is the number of blocks required. The blocks with gap fraction above 10% are not plotted.

Broad coverage of the pathogen population is particularly important when designing T-cell epitope vaccines against viral pathogens. Using FLAVIdB we applied the block entropy analysis method to the proteomes of the four serotypes of dengue virus (DENV) and found 1,551 blocks of 9-mer peptides, which cover 99% of available sequences with five or fewer unique peptides [35]. Many of the blocks are located consecutively in the proteins, so connecting these blocks resulted in 78 conserved regions which can be covered with 457 subunit peptides. Of the 1551 blocks of 9-mer peptides, 110 blocks consisted of peptides all predicted to bind to MHC with similar affinity and the same HLA restriction. In total, we identified a pool of 333 peptides as T-cell epitope candidates. This set could form the basis for a broadly neutralizing dengue virus vaccine. The results of block entropy analysis of dengue subtypes 1–4 from FLAVIdB are shown in Figure 7.

Influenza virus is a NIAID Category C Priority Pathogen. We developed the FLUKB that currently contains 302,272 influenza viral protein sequence entries from 62,016 unique strains (57,274 type A, 4,470 type B, 180 type C, and 92 unknown types) of influenza virus. It also catalogued 349 unique T-cell epitopes, 708 unique MHC binding peptides, and 17 neutralizing antibodies against hemagglutinin (HA) proteins along with their 3D structures. The detailed information on the neutralizing antibodies such as isolation information, experimentally validated neutralizing/escape influenza strains, B-cell epitope on the 3D structures, are also provided.

Approximately 10% of B-cell epitopes are linear peptides, while 90% are formed from discontinuous amino acids that create surface patches resulting from 3D folding of proteins [36]. Characterization of an increasing number of broadly neutralizing antibodies specific for pathogen surface proteins, the growing number of known 3D structures of antigen-neutralizing antibody complexes, and the rapid growth of the number of viral variant sequences demand systematic bioinformatics analyses of B-cell epitopes and cross-reactivity of neutralizing antibodies. We developed a generic method for the assessment of neutralizing properties of monoclonal antibodies. Previously, dengue virus was used to demonstrate a generalized method [27]. This methodology has direct relevance to the characterization and the design of broadly neutralizing vaccines.

Using the FLUKB, we employed the analytical methods to estimate cross-reactivity of neutralizing antibodies (nAbs) against surface glycoprotein HA of influenza virus strains, both newly emerging or the existing ones [26]. We developed a novel way of describing discontinuous motifs as virtual peptides to represent B-cell epitopes and to estimate potential cross-reactivity and neutralizing coverage of these epitopes. Strains labelled as potentially cross-reactive are those that share 100% identity of B-cell epitopes with experimentally verified neutralized strains. Two workflow types were implemented in the FLUKB for cross-neutralization analysis: cross-neutralization estimation workflow and B-cell epitope mapper workflow.

**Figure 8:**
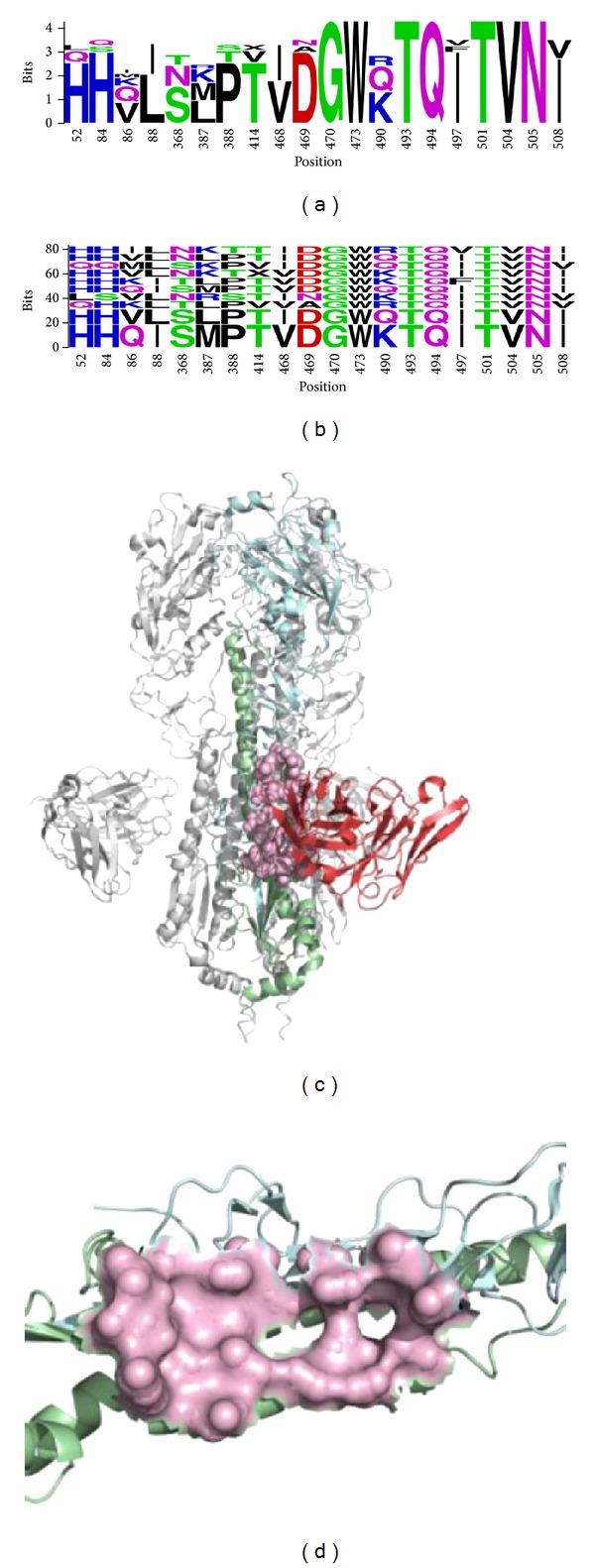
(a) Sequence logo of neutralizing epitopes by neutralizing antibody F10 on influenza virus HA protein. (b) BlockLogo of the discontinuous residues in F10 neutralizing epitope. (c) The structure of influenza A HA protein with neutralizing antibody F10 (PDB ID:3FKU) and the conformational epitope shown in pink. (d) Discontinuous epitope on HA protein recognized by F10.

The cross-neutralization estimation workflow estimates the cross-neutralization coverage of a validated neutralizing antibody using all full-length sequences of HA hosted in the FLUKB, or using full-length HA sequences of a user-defined subset by restricting year ranges, subtypes, or geographical locations. Firstly, a MSA is generated using the full-length HA sequences. The resulting MSA provides a consistent alignment position numbering scheme for the downstream analyses. Secondly, for each nAb, the HA sequence from its 3D structure and from the experimentally validated strains is used to search for a strain with the highest similarity in FLUKB using BLAST. Thirdly, a B-cell epitope is identified from the validated antigen-antibody structures based on the calculation of accessible surface area and atom distance. Fourthly, using the MSA and the alignment position numbering, the residue position of the B-cell epitope is mapped onto the HA sequences of validated strains to get B-cell epitope motifs. Discontinuous motifs are extracted from all the HA sequences in the MSA and compared to the B-cell epitope motif. According to the comparison results, they are classified to be either neutralizing if identical to a neutralizing discontinuous motif, escape if identical to an escape discontinuous motif, or not validated if no identical match was found. The cross-neutralization coverage estimation of neutralizing antibody F10 on all HA sequences from FLUKB is shown in Figure 8.

For a newly emerged strain, the B-cell epitope mapper workflow performs* in silico* prediction of its cross-neutralization based on existing nAbs and provides preliminary results for the design of downstream validation experiments. Firstly, a discontinuous peptide is extracted from its HA sequence according to positions on each known B-cell epitope. Secondly, sequence similarity comparison is conducted between the discontinuous motifs and all known B-cell epitopes from experimentally validated strains. The motifs identical to the known neutralized or escape B-cell epitope motifs are proposed as neutralized or escape strains, respectively.

The cross-neutralization estimation workflow provides an overview of cross-neutralization of existing neutralizing antibodies, while B-cell epitope mapper workflow gives an estimation of possible neutralizing effect of new viral strains using known neutralizing antibodies. This knowledge-based approach improves our understanding of antibody/antigen interactions, facilitates mapping of the known universe of target antigens, allows the prediction of cross-reactivity, and speeds up the design of broadly protective influenza vaccines.

## 4. Conclusions

The big data analytics applies advanced analytic methods to data sets that are very large and complex and that include diverse data types. These advanced analytics methods include predictive analytics, data mining, text mining, integrated statistics, visualization, and summarization tools. The data sets used in our case studies are complex and the analytics is achieved through the definition of workflow. Data explosion in our case studies is fueled by the combinatorial complexity of the domain and the disparate data types. The cost of analysis and computation increases exponentially as we combine various types of data to answer research questions. We use the* in silico* identification of influenza T-cell epitopes restricted by HLA class I variants as an example. There are 300,000 influenza sequences to be analyzed for T-cell epitopes using MHC binding prediction tools based on artificial neural networks or support vector machines [37–40]. Based on the DNA typing for the entire US donor registry, there are 733 HLA-A, 921 HLA-B, and 429 HLA-C variants, a total of 2083 HLA variants, observed in US population [41]. These alleles combine into more than 45,000 haplotypes (combinations of HLA-A, -B, and -C) [41]. Each of these haplotypes has different frequencies and distributions across different populations. The* in silico* analysis of MHC class I restricted T-cell epitopes includes MHC binding prediction of all overlapping peptides that are 9–11 amino acids long. This task alone involves a systematic analysis of 300,000 sequences that are on average 300 amino acids long. Therefore, the total number of* in silico* predictions is approximately 300,000 × 300 × 3 × 2083 (number of sequences times the average length of each sequence times 3 times the number of observed HLA variants) or a total of 5.6 × 10^11^ calculations. Predictive models do not exist for all HLA alleles, so some analysis needs to be performed by analysis of similarity of HLA molecules and grouping them in clusters that share binding properties. For B-cell epitope analysis, the situation is similar, except that the methods involve the analysis of 3D structures of antibodies and the analysis of nearly 100,000 sequences of HA and neuraminidase (NA) and their cross-comparison for each neutralizing antibody. A rich set of visualization tools is needed to report population data and distributions across populations. For vaccine studies, these data need to be analyzed together with epidemiological data including transmissibility and severity of influenza viruses [42]. These functional properties can be assigned to each influenza strain and the analysis can be performed for their epidemic and pandemic potential. These numbers indicate that the analytics methods involve a large amount of calculations that cannot be performed using brute force approaches.

Immunological knowledge discovery relies heavily on both the availability of accurate, up-to-date, and well-organized data and the proper analytics tools. We propose the use of knowledge-based approaches by developing knowledgebases combining well-annotated data with specialized analytical tools and integrating them into analytical workflow. A set of well-defined workflow types with rich summarization and visualization capacity facilitates the transformation from data to critical information and knowledge. By using KB-builder, we enabled streamlining of normally time-consuming process of database development. The knowledgebases built using KB-builder will speed up rational vaccine design by providing accurate and well-annotated data coupled with tailored computational analysis tools and workflow.
